# Relationship between musculoskeletal disorders, risk factors and sleep quality in healthcare workers

**DOI:** 10.1590/1806-9282.20250557

**Published:** 2025-10-27

**Authors:** Murat Doğan, Anıl Özüdoğru

**Affiliations:** 1 Kırşehir Ahi Evran University, Faculty of Medicine, Department of Family Medicine – Kırşehir, Türkiye.; 2 Kırşehir Ahi Evran University, School of Physical Therapy and Rehabilitation – Kırşehir, Türkiye.

**Keywords:** Musculoskeletal diseases, Risk factors, Sleep quality, Healthcare workers

## Abstract

**OBJECTIVE:**

Musculoskeletal disorders are highly prevalent among healthcare workers due to physically demanding tasks and stressful work conditions. These disorders contribute to decreased work performance, pain, and impaired quality of life. Sleep quality is an essential factor in overall health and has been linked to pain perception and musculoskeletal health. However, the relationship between musculoskeletal disorders and sleep quality remains unclear. The aim of this study was to investigate the prevalence of musculoskeletal disorders among healthcare workers, identify associated risk factors, and examine the relationship between musculoskeletal disorders and sleep quality.

**METHODS:**

This cross-sectional study was conducted in a state hospital in Kırşehir, Turkey, with 249 healthcare workers. Participants completed a sociodemographic questionnaire, the Cornell Musculoskeletal Disorders Questionnaire to assess musculoskeletal disorders, and the Pittsburgh Sleep Quality Index to evaluate sleep quality. Statistical analyses included Pearson's correlation test, t-test, and analysis of variance to examine associations between musculoskeletal disorders, sleep quality, and other risk factors.

**RESULTS:**

A significant relationship was found between musculoskeletal disorders and sleep quality (p<0.001). Healthcare workers with poor sleep quality reported higher Cornell Musculoskeletal Disorders Questionnaire scores. Additionally, musculoskeletal disorder prevalence was significantly associated with working hours (p=0.018) and income level (p=0.047). Nurses had the highest musculoskeletal disorder scores among occupational groups.

**CONCLUSION:**

Musculoskeletal disorders in healthcare workers are influenced by sleep quality, working hours, and income level. Ergonomic interventions and policies to improve sleep quality may help reduce musculoskeletal disorder prevalence and enhance overall well-being in this workforce.

## INTRODUCTION

Research in the field of occupational health has identified various physical and psychosocial risk factors that cause occupational musculoskeletal disorders (MSDs). These studies show that physically demanding work practices such as lifting or carrying heavy loads, tiring positions, awkward postures, and repetitive movements, as well as stressful working conditions, directly lead to MSDs^1^.

Healthcare workers such as nurses, midwives, surgeons, physiotherapists, and dentists are at a high risk of musculoskeletal problems due to their intense work tempo, repetitive movements, standing for long periods, lifting heavy loads, and non-ergonomic working positions. Studies show that the prevalence of MSDs exceeds 80% in these groups^2,3^. Pain, especially in the low back, neck, shoulder, and knee areas, can lead to loss of work power, decreased professional performance, and deterioration of quality of life^4^.

Sleep is a universal function in all living species, accounting for approximately one-third of human life. Insufficient or poor-quality sleep can cause significant disorders across multiple systems, including the endocrine system, metabolism, cognitive functions, and the nervous system^5,6^.

It is observed that pain and sleep disorders often occur together^7^. However, the causal relationship between these two conditions has not yet been clearly defined^8^. Prospective studies examining the relationship between sleep and pain show that sleep disorders can increase the severity of pain and that pain can negatively affect sleep quality^9^.

Factors such as intense work, ergonomic inadequacy, and shift work create both pain and discomfort in the musculoskeletal system and disrupt sleep patterns, reducing the quality of rest. This situation can negatively affect the general health and work efficiency of healthcare workers. The aim of this research was to determine MSDs and risk factors in healthcare workers and to reveal their relationship with sleep quality.

## METHODS

This study was conducted with hospital staff in a state hospital in Kırşehir province. It was determined by the decision of the local ethics committee that there was no ethical problem in conducting the study (139-17.09.2024). After the participants read, understood, and approved the informed consent form, those who met the inclusion criteria were assessed for sociodemographic information, musculoskeletal symptoms, and sleep quality.

The sociodemographic information of the participants was evaluated with the survey prepared by the researchers, musculoskeletal symptoms were evaluated using the "Cornell Musculoskeletal Disorders Questionnaire," (CMDQ) and sleep quality was evaluated using the Pittsburgh Sleep Quality Index (PSQI).

The sample size was calculated to be 10 times the number of questions in the survey with the highest number of questions among the surveys used in the study, as suggested by Bryman and Cramer^10^. The survey with the highest number of questions in our study was the "CMDQ" and the number of questions was 20. Accordingly, the minimum sample size was found to be 200, based on 10 times the 20 survey questions. By including additional reserve recruitments, 249 people were recruited for our research.

### Cornell Musculoskeletal Disorders Questionnaire

The CMDQ is a measurement tool developed to assess MSDs. The Turkish validity and reliability study was conducted in 2008. The questionnaire evaluates the frequency, severity, and impact of pain, aches, or discomfort in the last week. A total of 20 body regions are scored separately, and the total discomfort score ranges from 0 to 90. When calculating the score, frequency, severity, and work disability are multiplied. As the CMDQ score increases, the frequency, severity, and negative impact of pain on work also increase^11^.

### Pittsburgh Sleep Quality Index

The PSQI was used to assess sleep quality. This scale allows sleep to be determined as good or bad and to be evaluated quantitatively. The survey consists of a total of 24 questions, 19 of which are answered by the individual and five by the roommate, but this part is not included in the scoring. The scale evaluates seven components: subjective sleep quality, sleep latency, sleep duration, sleep efficiency, sleep disturbances, use of sleeping pills, and daytime dysfunction. The total score ranges from 0 to 21. Scores of five and below are considered good sleep quality, and scores above five are considered poor sleep quality^12,13^.

### Statistical analysis

The general characteristics of the study population were calculated as a percentage distribution. The relationship between continuous variables and MSDs was evaluated using Pearson's correlation analysis. Whether there was a significant difference in terms of MSDs in categorical variables was analyzed with analysis of variance (ANOVA) and t-test. The significance level was accepted as p<0.05.

## RESULTS

A total of 249 healthcare workers, 106 males and 143 females, participated in the study. General characteristics of the study population are shown in Table 1. Among the continuous variables, only working hours were found to be significantly associated with MSDs. In addition, a significant relationship was found between MSDs and all components of sleep quality (except habitual sleep efficiency) (Table 2). When evaluating whether there was a significant difference in terms of MSDs in categorical variables, it was seen that there was a significant difference in income level and sleep quality. Accordingly, the prevalence of MSDs was significantly higher in individuals with low income levels compared to those with middle income levels. In addition, MSDs were significantly higher in participants with low sleep quality compared to those with good sleep quality (Table 3).

**Table 1:** General characteristics of the study population.

Variable	Category	n	%
Gender	Male	106	42.6
	Female	143	57.4
Marital status	Single	75	30.1
	Married	171	68.7
	Widowed	3	1.2
Education level	Primary	1	0.4
	Secondary	1	0.4
	High school	32	12.9
	Higher education	204	81.9
	M.Sc./Ph.D.	11	4.4
Number of children	0	100	40.2
	1	43	17.3
	2	75	30.1
	3	25	10.0
	4	6	2.4
Occupation	Driver	19	7.6
	Paramedic	121	48.6
	Doctor	11	4.4
	Technician	19	7.6
	Nurse	44	17.7
	Office worker	31	12.4
	Worker	4	1.6
Employment type	Daytime	79	67.9
	On duty	170	30.9
Income level (TL)	Low	169	67.9
	Medium	77	30.9
	High	3	1.2
Smoking	Absent	169	67.9
	Present	80	32.1
Alcohol	Absent	203	89.8
	Present	23	10.2
Exercise habit	Absent	180	72.3
	Present	69	27.7
Exercise type	Resistive	13	5.2
	Aerobic	38	15.3
	Resistive+aerobic	4	1.6
Chronic disease	Diabetes	14	21.9
	Hyperlipidemia	3	4.7
	Anemia	14	21.9
	Hypertension	12	18.8
	COPD	2	3.1
	CAD	2	3.1
	Others	19	29
Occupational positions	Heavy lifting^a^	40	16.1
	Bending forward^b^	32	12.9
	Sitting for long periods^c^	56	22.5
	Standing for long periods^d^	40	16.1
	a+b	17	6.8
	a+b+c	33	13.3
	a+b+d	31	12.4

n: number of participants; COPD: chronic obstructive pulmonary disease; CAD: coronary artery disease.

a heavy lifting;

b bending forward;

c sitting for long periods;

d standing for long periods.

**Table 2:** Correlations between continuous variables and Cornell Musculoskeletal Disorders Questionnaire scores.

Variables		r	p
Age		-0.06	0.337
Height		-0.11	0.109
Weight		-0.01	0.709
BMI		0.03	0.616
Year of profession		-0.07	0.273
Duration of work		**-0.16**	**0.018** *
Smoke consumption		0.10	0.125
Exercise habit duration		-0.06	0.638
Exercise frequency (day/week)		-0.08	0.564
Exercise duration (min/day)		0.07	0.613
Number of steps		0.06	0.372
Sleep quality			
	Subjective sleep quality	0.22	**0.001** *
	Sleep latency	0.24	**0.001** *
	Sleep duration	0.20	**0.003** *
	Habitual sleep efficiency	0.02	0.774
	Sleep disturbances	0.373	**<0.001** *
	Use of sleeping medications	0.154	**0.028** *
	Daytime dysfunction	0.22	**0.001** *
	Total sleep quality	0.33	**<0.001** *

* p<0.05, r: correlation coefficient; BMI: body mass index. Statistically significant values are denoted in bold.

**Table 3:** Comparison of musculoskeletal disorders across categorical variables.

Variable	Category	CMD score (mean±SD)	T or F statistic	p
Gender	Male	21.53±50.75	-2.316	**0.022** *
	Female	51.40±128.75		
Marital status	Single	28.57±50.52	0.563	0.570
	Married	44.95±123.107		
	Widowed	16.66±15.27		
Education level	High school	15.64±37.66		
	Higher education	43.73±115.917	0.509	0.729
	M.Sc./Ph.D.	80.80±70.51		
Number of children	0	35.07±70.22	0.506	0.731
	1	39.00±154.48		
	2	38.04±14.72		
	3	68.04±132.98		
	4	9.75±11.32		
Occupation	Driver	8.00±22.06	1.245	0.285
	Paramedic	33.47±113.54		
	Doctor	24.42±35.46		
	Technician	31.55±53.98		
	Nurse	76.39±145.44		
	Office worker	33.80±73.66		
	Worker	9.50±19.00		
Employment type	Daytime	47.53±87.90	0.817	0.447
	On duty	35.60±115.10		
Income level (TL)	Low^a^	28.17±79.46	2.970	**0.047** * (a and b)
	Medium^b^	66.42±147±99		
	High^c^	10.00±17.32		
Smoking	Absent	34.82±95.43	-0.946	0.345
	Present	49.83±125.73		
Alcohol	Absent	41.08±112.05	0.355	0.723
	Present	32.33±39.15		
Exercise habit	Absent	36.26±92.00	-0.743	0.458
	Present	48.55±136.23		
Exercise type	Resistive	28.22±38.97	0.554	0.579
	Aerobic	72.60±177.93		
	Resistive+aerobic	4.5.±6.60		
Chronic disease	Diabetes	40.00±85.72	1.458	0.221
	Hyperlipidemia	6.00±8.48		
	Anemia	127.72±268.01		
	Hypertension	151.22±215.28		
	COPD	-		
	CAD	300.00±424±26		
	Others	44.70±85.64		
Occupational positions	Heavy lifting	6.89±23.35	1.509	0.215
	Bending forward	45.23±60.41		
	Sitting for long periods	30.15±96.29		
	Standing for long periods	48.39±110.88		
Sleep quality	Low^a^	81.36±121.98	3.658	**0.037** * (a–c)
	Medium^b^	46.21±118.56		
	High^c^	16.50±70.57		

* p<0.05, CMD: Cornell Musculoskeletal Disorders Questionnaire; COPD: chronic obstructive pulmonary disease, CAD: coronary artery disease; SD: standard deviation; TL: Turkish liras. Statistically significant values are denoted in bold.

a low;

b medium;

c high.

## DISCUSSION

This study, which was conducted to examine the factors affecting MSDs in healthcare workers and the relationship between MSDs and sleep quality, found that MSDs in healthcare workers were related to working hours and sleep quality. It was also found that healthcare workers showed significant differences in terms of MSDs according to their income levels and sleep quality levels. Numerous studies in the literature have examined MSDs among healthcare workers. For example, according to the study conducted by Zaheer et al., the highest prevalence of work-related musculoskeletal problems was found among medical technologists, followed by nurses^14^. Another study found that nurses and doctors have higher rates of MSDs than other healthcare professions^15^. Similarly, our study found that nurses are prone to musculoskeletal problems. According to the findings of the study, it was determined that musculoskeletal problems differ between occupational groups. This situation can be explained by the fact that these occupational groups are physically more strained. In addition, a significant relationship was found between working hours and musculoskeletal problems in our study. This is an expected result considering the working hours and increasing workload.

A 7-year cross-sectional study by Pan et al. found that low-income individuals had poorer musculoskeletal health than high-income individuals^16^. In another study, the primary sociodemographic factors affecting MSDs of homemakers were found to be household income and body mass index. In this study, a significant difference was observed between income level and MSDs. It was determined that individuals with low income levels had more MSDs. This situation may be related to the fact that individuals with low income levels work under more challenging physical conditions and benefit less from ergonomic conditions. However, more research is needed to explain the causal relationship between MSDs and income level.

One of the most striking findings of this study is the relationship between MSDs and sleep quality. The findings showed that individuals with poor sleep quality were significantly more likely to have MSDs. The findings of a systematic review and meta-analysis covering 16 articles and 11 different study populations provide evidence, albeit with very low certainty, that baseline sleep problems/disorders are a risk factor for chronic musculoskeletal problems in both the short and long term^17^. Another study showed that nurses’ sleep duration, time to fall asleep, and sleep quality significantly contributed to the development of neck and upper back pain^18^. Although sleep quality is largely assessed subjectively, it is known that sleep disorders are more common, especially in individuals who work shifts. It can be said that a significant portion of healthcare professionals also work shifts. The fact that sleep disorders are associated with MSDs can be attributed to the body's regeneration and repair processes being affected. Sleep has a critical role in the repair of muscle tissue and general well-being. In addition, it is thought that sleep disorders cause an increase in inflammatory markers, which in turn affects chronic pain mechanisms. It is known that sleep insufficiency lowers the pain threshold and increases the perception of MSDs^9^.

The findings of this study reveal the necessity of ergonomic arrangements and improvements in the work environment to reduce MSDs in healthcare workers. Workload adjustments and the use of appropriate supportive equipment are recommended, especially for high-risk groups such as nurses and paramedics.

In addition, work order improvements that will increase sleep quality are also of great importance. Rearranging shift hours can help healthcare workers optimize their sleep cycles.

In conclusion, it has been shown that MSDs in healthcare workers are affected by both individual and environmental factors and that sleep quality plays an important role in these disorders. Therefore, strategies to improve these two factors are critical to improving the overall health and work performance of healthcare workers.

## Data Availability

The datasets generated and/or analyzed during the current study are available from the corresponding author upon reasonable request.
